# Activated Sintering of Cr_2_O_3_-Based Composites by Hot Pressing

**DOI:** 10.3390/ma15175960

**Published:** 2022-08-29

**Authors:** Edwin Gevorkyan, Lenka Cepova, Mirosław Rucki, Volodymyr Nerubatskyi, Dmitrij Morozow, Wojciech Zurowski, Voskan Barsamyan, Karel Kouril

**Affiliations:** 1 Faculty of Mechanics and Energy, Ukrainian State University of Railway Transport, 7 Feuerbach Sq., 61050 Kharkiv, Ukraine; 2 Institute of Mechanical Science, Vilnius Gediminas Technical University, J. Basanaviciaus Str. 28, LT-03224 Vilnius, Lithuania; 3 Faculty of Mechanical Engineering, VSB-Technical University of Ostrava, 17. listopadu 2172/15, 70800 Ostrava Poruba, Czech Republic; 4 Faculty of Mechanical Engineering, Kazimierz Pulaski University of Technology and Humanities in Radom, Stasieckiego 54, 26-600 Radom, Poland; 5 National Polytechnic University of Armenia, Vanadzor 2011, Armenia; 6 Faculty of Mechanical Engineering, Brno University of Technology, Technicka 2896/2, 61669 Brno, Czech Republic

**Keywords:** composite, chromium oxide, reactive sintering, solid solution, phase boundaries

## Abstract

The paper presents and discusses questions on structure formation during the sintering process of Cr_2_O_3_-based composites using the hot pressing method, when a chemical reaction between the components takes place. The task was difficult because Cr_2_O_3_ decomposes when sintered at temperatures above 1300 °C. The proposed novel method allowed for interaction between aluminum and chromia, thus avoiding the decomposition of the latter. Here, ultrafine aluminum powder played the role of the active agent forming a liquid phase and reacting with Cr_2_O_3_. The appearance of the solid solutions of (Cr,Al)_2_O_3_ with different stoichiometry of Cr and Al depended on the aluminum content in the initial mixture. The solid solution significantly strengthened boundaries between composite phases, resulting in the composite material of high fracture toughness between 5 and 7 MPa m^½^ and bending strength of ca. 500 MPa. The best mechanical properties exhibited the cermet with 22 wt.% of the restored chromium.

## 1. Introduction

Metal oxides are very important for the composite industry. In ceramic matrix composites, they form the matrix phase, providing high thermal and chemical stability, high compressive strength, high elastic modulus and other advantageous properties [1].

Alumina-based ceramics are popular materials for cutting tools due to their good wear resistance, high hardness, heat resistance and chemical stability [2]. There are many reports on the effects of various reinforcements, such as graphene [3], SiC [4,5], SiC_w_-Si_3_N_4_ [6], TiC [7], TiC-TiN [8] and WC [9], and the works on property improvement are still going on. Basically, sintering methods are used for the fabrication of ceramic matrix composites [10,11,12]. Recently, the addition of amorphous phases was found useful for preparing micro/nano bimodal microstructures [13].

Compared to aluminum oxide (Al_2_O_3_), chromia has higher hardness and melting temperature, so ceramic composites based on chromium oxide (Cr_2_O_3_) can be expected to be advantageous for cutting tool applications. It was initially demonstrated that these composites provided a high quality of the machined hard steel surfaces [14]. The application of chromium oxide based composites for the machining of high-hardness steels seems to be advantageous compared to the traditional, non-regrindable cutting inserts made from aluminum oxide (Al_2_O_3_) and silicon nitride (Si_3_N_4_). Especially beneficial is the increase in machined surface quality, which in some conditions even provides accuracy after reaching close to that obtained after grinding. However, chromia is rather added to the alumina composites to enhance their properties [15], with rare attempts to sinter a Cr_2_O_3_-based composite. This is because of the difficulties in obtaining sintered chromia-based dense composite due to the decomposition of Cr_2_O_3_ at temperatures above 1300 °C. 

Cr_2_O_3_ is a hexagonal crystal with a melting point of 2435 °C and a high Vicker’s hardness of 1825 HV [16]. Chromium oxide (Cr_2_O_3_) is widely known as a catalyst [17], but it also exhibits high abrasive properties and thus has been widely used in form of polishing pastes. It is an important refractory material with high melting temperature and high oxidation resistance at elevated temperature [18]. Chromia coatings have been widely applied for wear- and corrosion-resistant components in different sectors of industry [19]. Moreover, chromia coatings exhibit high mechanical strength and high microhardness [20]. Since most of the mechanical characteristics of chromia are better than those of alumina, a Cr_2_O_3_-based composite for cutting tools can be expected to be advantageous, if it would be possible to avoid the decomposition of chromia during sintering.

The addition of chromia to alumina provides the alumina–chromia solid solutions that are widely used in many applications where high-temperature corrosion resistance is required [21]. From the perspective of cutting tool requirements, a composite Cr_2_O_3_–22 wt.% Cr is the most interesting, due to the appearance of eutectics of finely dispersed structure [22]. In this structure, small spherical chromium reinforcement particles are distributed within the Cr_2_O_3_ matrix. 

The present study is devoted to the sintering of a chromia-based composite with an aluminothermic reaction, where the metallic chromium is released. The objective is to calculate the respective proportion of the initial components leading to a proper eutectic composition after sintering that results in advantageous properties of the sintered Cr_2_O_3_-based composite.

## 2. Materials and Methods

**Figure 1:**
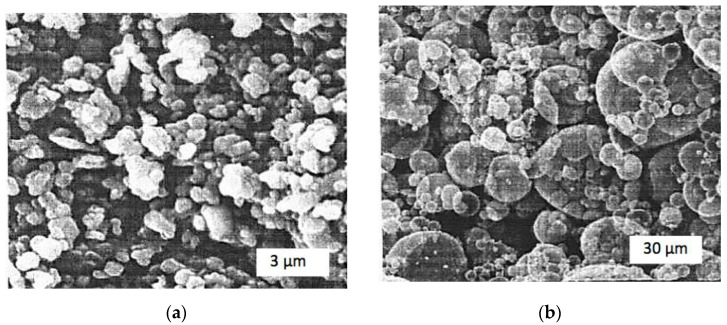
SEM images of the initial powders: (**a**) Cr_2_O_3_; (**b**) Al.

Initial powders used for the experiments were finely crushed, ultradispersed ones. Cr_2_O_3_ powder delivered by Sigma Aldrich Chemie GmbH (Taufkirchen, Germany) had particle dimensions between 1 and 3 μm, while the aluminum powder type PAP-2, made by PromMetal (Moscow, Russia), had particles in form of small plates from several microns to 30 μm large. The SEM images of the powders are shown in Figure 1.

The powders were mixed together in the agate drums using a planetary ball mill, at a speed of 5 m/s for 30 min in the environment of ethanol. The proportion of the powder mixture, ethanol and mixing balls was 2:1:1. After the procedure, the mixtures were dried and rubbed through a #0064 sieve.

The powder mixture of chromium oxide and aluminum was then briquetted in steel molds in a vacuum. These samples then underwent a hot pressing procedure in a vacuum of 10^−2^ Pa under a pressure of 30 MPa in the electric field.

In order to investigate the interaction between the powders of Cr_2_O_3_ and aluminum in the mixture, differential thermal analysis (DTA) was employed. In the original device, a patented high-temperature unit was installed, enabling analysis of the thermal effects of chemical reactions [23]. For that purpose, a powder mixture of Cr_2_O_3_ + 13 wt.% Al was prepared.

The physical and mechanical characteristics of the tested composites were determined with standard methodology and devices. The flexural strength was measured using an MH-1 machine with samples of dimensions 5 × 5 × 35, base 30 mm. Concentrated force was applied at a velocity of 40 m/s, and in one pack, there were 5 samples.

Compressive strength was measured using the standard device UMN-10 produced by Alexandrov Factory (Alexandrovka, Russia). The respective samples were ground until the methodologically recommended parameters [24] were reached. Five samples of dimensions 5 × 5 × 10 mm were used for one point. The Microhardness of the composites was measured using a PMT 3 GEO-NDT device (Russia) with optimal loads and time of measurement.

The fracture toughness *K_IC_* was determined from the indentation with Vickers’ pyramid on the polished surfaces according to the following formula [25,26]:

(1) $$K_{IC}\cdot F\left(H\cdot a^{\frac{1}{2}}\right)\cdot {\left(\frac{H}{E\cdot F}\right)}^{\frac{2}{5}}=0.35\cdot {\left(\frac{l}{a}\right)}^{-\frac{1}{2}},$$

where F is the constraint factor, F = 3; *H* is the hardness; *a* is the half-diagonal of the Vickers indent; *E* is the Young’s modulus; and *l* is the length of the surface crack. The dimensions of samples made for that purpose were 13 mm × 13 mm × 5 mm. 

Relative density *D_r_* was calculated using the following equation:

(2) $$D_{r}=\frac{D_{b}}{D_{th}}\cdot 100\;\%,$$

where *D_b_* is the actual density of a sintered specimen in g/cm^3^ and *D_th_* is the theoretical density of the solid material in g/cm^3^.

XRD spectra of the powders were measured using Dron-3.0 and URS-50 diffractometers with Cu-Kα radiation and Ni filter.

A fractography study was carried out using a scanning electron microscope (SEM) JEOL JSM-840 (JEOL, Tokyo, Japan). Micro XRD analysis was performed with a CamScan series SEM, which enabled the assessment of the qualitative, semi-quantitative and quantitative distribution of the components on the surface points. Qualitative chemical analysis along the profile was performed with the Link-860 energy-dispersive spectroscopy (EDS) system (Link Systems Ltd., High Wycombe, UK), while the ZAF-4 correction factor was used for the quantitative determination of chemical composition. Semi-quantitative assessment was performed using Digimap proprietary software. All the results underwent statistical analysis.

A metallographic study of the obtained structures was performed using the MIM-8 (LOMO, St. Petersburg, Russia) microscope in reflected light, with a zoom from 300× to 1000×. For that purpose, the samples were ground with diamond discs of 60/40, 20/14, 7/5 and 3/2 μm grains with a coolant in form of water emulsion.

## 3. Theoretical Background

According to the main goal of the research, it is crucial to determine the initial proportion of powders in order to find the final proportion of components preventing the decomposition of chromia. It is known that in the composite structure, chromium can be obtained from the chromium oxide through the aluminothermic reaction [27]:

(3) $${\mathrm{Cr}}_{2}O_{3}+2\mathrm{Al}=2\mathrm{Cr}+{\mathrm{Al}}_{2}O_{3}.$$

From this equation, it is seen that by varying the aluminum content in the initial powder, it is possible to fabricate composite materials of different compositions and stoichiometry and thus with different properties. Among other materials, it is possible to obtain the abovementioned eutectic composition indicated in [22] (see Section 1). A simple assessment indicates that the target 22 wt.% chromium will be restored when the initial chromia powder contains 13 wt.% aluminum. When processing the powder mixture of Cr_2_O_3_ + 13 wt.% Al, it was expected to obtain the final products of reaction with the required eutectic proportion of chromium: Cr_2_O_3_ + (Cr,Al)_2_ + 22 wt.% Cr.

It is a known fact that the effectiveness of this process is dependent on the particle dimensions of both metal oxide and aluminum, as well as on the mixture composition. Apparently, the finer the powder is, the more intense the reaction is.

## 4. Results and Discussion

### 4.1. Effect of Sintering Conditions on Relative Density

**Figure 2:**
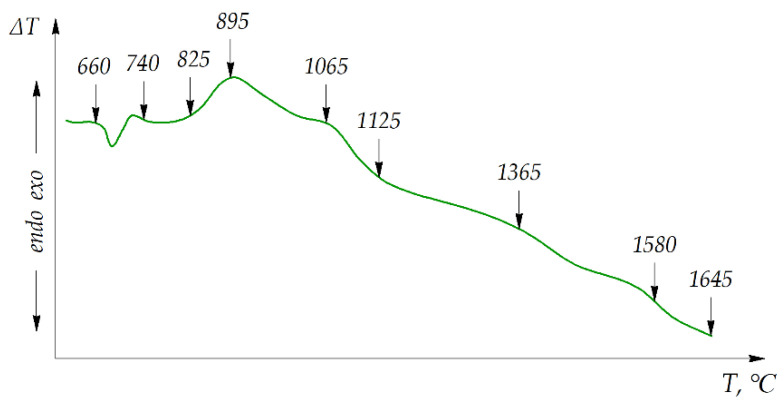
Thermogram of the initial powder mixture Cr_2_O_3_ + 13 wt.% Al.

The DTA curve shown in Figure 2 demonstrated that the interaction between chromium oxide and aluminum started at 825 °C. A substantial exothermal effect took place at 895 °C, while the presence of the exothermal effect at 660 °C was related to the initiation of the aluminum powder melting process.

Thermal equilibrium can be described by the following equation [28]:

(4) $$\alpha \cdot \left(T_{a}–T_{s}\right)=m_{s}\cdot C\cdot n_{s}\cdot V+q\cdot \left(\frac{dm_{s}}{dt}\right)+m_{0}+C_{0}\cdot V+q_{loss},$$

where α is the coefficient of proportion, *T* is temperature, m is mass, *C* is specific heat capacity, *q* is specific enthalpy of tranformation and qloss is heat loss; *a*—ambient, *s*—sample, *o*—outer surface.

When the aluminum content in the mixture was 13 wt.%, the specific heat released during the reaction was *Q* = 925 J/kg. It was found that heat release for the mixtures with lower aluminum amounts was smaller; e.g., the mixture Cr_2_O_3_ + 13 wt.% Al provided *Q* = 712 J/kg. Hence, the sintering temperature for a higher Al proportion is higher.

Equilibrium parameters for the thermodynamical system were found from the determined extrema of the system’s entropy. Equilibrium concentration *C_e_* of the components for the cermet sintered from the mixture Cr_2_O_3_ + 13 wt.% Al was calculated for the temperature *T* range from 700 °C to 1400 °C and the pressure *P* range from 30 MPa to 0.01 MPa. The results of the calculations are shown in Table 1.

From Table 1 it can be seen that under pressure *P* = 30 MPa, the increased temperature caused variations in the concentrations of the components; in particular, chromium concentration increased while Cr_2_O_3_ and Al concentrations decreased. When the pressure was very low, *P* = 0.01 MPa, chromium content also increased with the increase in temperature, while aluminum practically disappeared. However, the calculated equilibrium concentration did not change substantially in the temperatures around 900–1000 °C and 1000–1400 °C at the pressure *P* = 30 MPa.

Hence, the calculations suggested that the sintering in temperature interval between 1300 °C and 1400 °C under the pressure of 30 MPa would result in no aluminum among the final products. Thus, these conditions ensure that the restoration reaction is fully completed. Moreover, it is possible to control, to some extent, the composition of the sintered sample with alteration of temperature and pressure.

**Figure 3:**
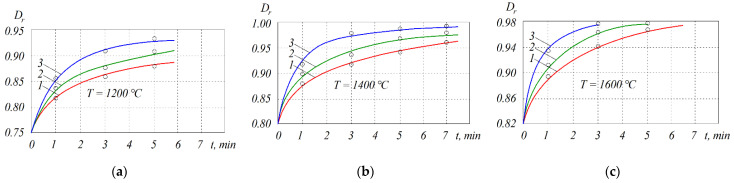
Interpolated curves of relative density *D_r_* of the Cr_2_O_3_ + (Cr,Al)_2_O_3_ + 22 wt.% Cr cermets sintered at different temperatures: (**a**) *T* = 1200 °C; (**b**) *T* = 1400 °C; (**c**) *T* = 1600 °C. Plots are denoted as follows: *1*—*P* = 15 MPa; *2*—*P* = 22 MPa; *3*—*P* = 30 MPa.

It should be noted that other processes such as viscous flow and diffusion take place during hot pressing. Figure 3 demonstrates that at *T* = 1600 °C, almost full density can be reached after 2 and 4 min under pressure *P* = 30 MPa and 22 MPa, respectively. Intense compaction can be attributed to the appearance of the liquid phase of molten aluminum above 660 °C, as well as exothermal reaction temperature increase at 895 °C.

In the experiments, the heating rate was ca. 400 °C/min. The pressure was applied gradually, dependent on the temperature of the graphite molds. After *T* = 660 °C was reached, the pressure was increased to the maximal value set for the particular experiment. This way the liquid aluminum was distributed steadily in the volume of the sintered cermet.

It should be noted that the densification rate slowed down. This can be attributed to the increase in the volume viscosity of the porous body and subsequent slowing down of the volume deformation rate.

### 4.2. Effect of Solid Solution

XRD analysis revealed the presence of a solid solution of (Cr,Al)_2_O_3_* in the sintered samples, apart from Cr_2_O_3_ and restored Cr. Results of the measurements of lattice parameters for different proportions of aluminum in the initial powders are shown in Table 2.

These results are in conformity with those of [29], which indicated the appearance of a solid solution between Cr_2_O_3_ and Al_2_O_3_ in a certain range of temperature. Oxides of chromium and aluminum have a corundum structure, where ions of oxygen are almost densely packed with ions of Al^3+^ or Cr^3+^. These occupy two-thirds of the available octahedral positions; thus, the obtained solid solution can be described as Al_2–*x*_Cr*_x_*O_3_ (0 ≤ *x* ≤ 2). For the in-between values of *x*, Al^3+^ and Cr^3+^ ions are distributed in octahedral positions disorderly. In a single octahedral cluster, ions of Al^3+^ and Cr^3+^ can be found, and the probability of a particular ion’s presence depends on the parameter *x*.

### 4.3. Composition of the Obtained Cermets

**Figure 4:**
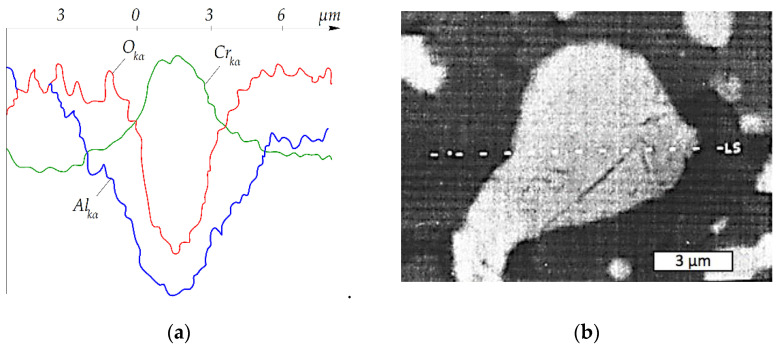
Qualitative distribution of the elements at the phase boundaries in the Cr_2_O_3_ + (Cr,Al)_2_O_3_ + 22 wt.% Cr sample sintered out of Cr_2_O_3_ + 13 wt.% Al powder at *T* = 1400 °C and *P* = 30 MPa for *t* = 10 min: (**a**) diagram; (**b**) SEM image of the analyzed sample.

Results of qualitative analysis of component distribution at the phase boundaries indicate the presence of some sort of transitional layer ca. 2 μm thick. Figure 4a presents a plot for the Cr_2_O_3_ + (Cr,Al)_2_O_3_ + 22 wt.% Cr composite made along the line marked LS in Figure 4b, across the grain. The sample was sintered at *T* = 1400 °C and *P* = 30 MPa for *t* = 10 min.

The distribution of elements in the boundary between the ceramic and metal compounds of the composite and the existence of a single phase of ceramic near the metal suggest that the transition zone consists of solid solution Al_2–*x*_Cr*_x_*O_3_. Both literature [29] data and XRD analysis confirmed this assumption. The presence of pure chromium in the gray grain confirmed its full recovery from Cr_2_O_3_, according to the expectations and objectives of the experiments.

Moreover, results of the quantitative chemical analysis suggested that the composition of Al_2–x_Cr_x_O_3_ solid solution depended not only on the aluminum amount, but also on the temperature of hot pressing and on the exposure time *t*. Table 3 presents the content of chromium and aluminum in the sintered samples of Cr_2_O_3_ + (Cr,Al)_2_O_3_ + 22 wt.% Cr, where the initial composition of powders Cr_2_O_3_ + 13 wt.% Al was the same but sintering time *t* was different.

Further XRD analysis revealed that the oxide lattice was substantially deformed. Namely, it formed a Cr_2_O_3_ structure of rhombohedral space group R3c with coherent addition of complex (Cr,Al)_2_O_3_ of hereditary structure. From Table 2 above, it can be seen that the aluminum content percentage in the initial powder did not affect the lattice parameters *a*_3_ and *c*_3_. From this fact, it can be concluded that these parameters are related to the chromium oxide lattice.

However, XRD showed that in the solid solution made out of the same composition, the lattice parameters *a*_1_, *a*_3_, *c*_1_ and *c*_3_ are varying for different sintering times. These variations perhaps can be attributed to the concentration of the ions Cr^3+^ and Al^3+^, which causes a difference between two micro areas rich in aluminum oxide and chromium oxide, respectively. Despite that, macroscopic analysis suggests the homogeneous structure of the solid solution. As a result, there are two different lattice structures with different parameters in the same solid solution. When the proportion of the components in the powder mixture is changed, the lattice parameters change too. In fact, an increase in Al proportion caused a decrease in the a parameter and an increase in the *c* parameter. Since the ratio *c*/*a* increases, the electron structure changes.

**Figure 5:**
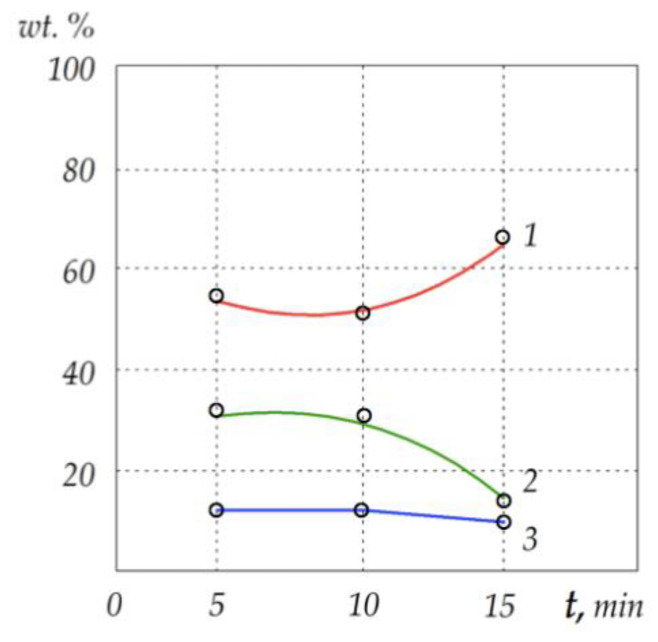
Percentage by weight of components in the dark phase of Cr_2_O_3_ + (Cr,Al)_2_O_3_ + 22 wt.% Cr cermet samples sintered at *T* = 1300 °C under *P* = 30 MPA for different lengths of time *t*. The curves are marked as follows: *1*—chromium; *2*—aluminum; *3*—oxygen.

Figure 5 shows the plots of chromium, aluminum and oxygen content in the sintered samples of Cr_2_O_3_ + (Cr,Al)_2_O_3_ + 22 wt.% Cr after different sintering times. It is clear that the proportion of chromium increased for longer sintering times, while that of aluminum decreased, which led to the variation in the composition of the solid solution. The experimental results suggest that the interaction of both oxides at the boundaries between the phases of oxide and metal provides a solid solution of Al_2–*x*_Cr*_x_*O_3_ (0 ≤ *x* ≤ 2) of varying composition. The quantitative proportion of the compounds in this solution depends on the initial powder composition, as well as on the sintering conditions, above all the temperature and sintering time.

### 4.4. Strength Analysis

**Figure 6:**
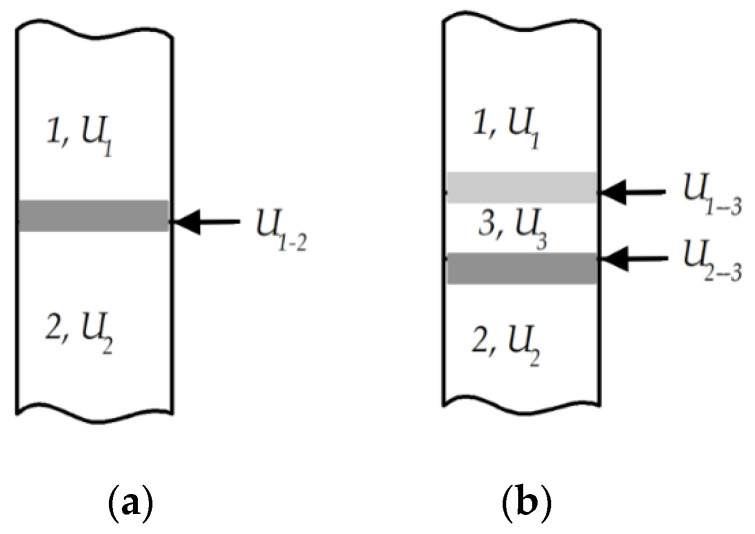
The simplified scheme of contact areas: (**a**) contact area directly between the metal (*1*) of strength *U*_1_ and oxide (*2*) of strength *U*_2_; (**b**) presence of the transitional phase (*3*) of strength *U*_3_ between the metal (*1*) and oxide (*2*).

It is widely known that the strength of MeO–Me cermets is determined by its weakest element, namely the bond of the ceramic/metal interface [30]. The solid solution Al_2–*x*_Cr*_x_*O_3_ resulting from an aluminothermic reaction seems to be the weakest point of the tested composite since it represents an intermediate phase and determines the strength of metal–oxide contact. The simplified scheme of the contact area between the metal (*1*) and oxide (*2*) is presented in Figure 6a, and the presence of the transitional phase in presented Figure 6b. An increase in the composite strength is possible when

(5) $$U_{1-2}<U_{2};U_{1-2}<U_{3};U_{1-2}<U_{1-3},$$

where *U*_1_, *U*_2_ and *U*_3_ denote the strengths of metal, oxide and transitional phases, respectively, while *U*_1–2_, *U*_1–3_ and *U*_2–3_ denote the strengths of the boundaries between the phases [27].

From the literature, it can be found that chromium oxide is more active than oxides Al_2_O_3_ and ZrO_2_ in interaction with metals [31]. This can be attributed to the basic properties of the metal oxides [32], since the thermodynamic stability of Cr_2_O_3_ is lower than that of Al_2_O_3_ and ZrO_2_, while the pressure of its dissociation products is higher than that of Al_2_O_3_ and ZrO_2_.

During the hot pressing, aluminum melts at the temperature of 660 °C, introducing a liquid phase to the sintered powders. A liquid phase usually serves as a sintering aid for the ceramic matrix composite [33]. The structure and, hence, the mechanical properties of the sintered cermet depend largely on the distribution of the aluminum throughout the sample volume. The molten aluminum wets the solid surface of chromium oxide and starts to interact with it at 825 °C.

Work of adhesion *W_a_* can be calculated from the known equation [34]:

(6) $$W_{a}=\gamma_{A}+\gamma_{B}-\gamma_{AB},$$

where *γ_A_* and *γ_B_* are the surface energies of the two substances being bonded and *γ_AB_* is interfacial energy between phases A and B.

Moreover, since van der Waals forces are in the nanometer range, they are responsible for contact interactions [35], which can be included in the following equation:

(7) $$W_{a}=W_{b}+W_{ch},$$

where *W_b_* is van der Waals interaction energy and *W_ch_* is the energy of chemical interaction.

The equilibrium of liquid aluminum on the solid surface of chromium oxide can be described by the so-called Young equation [36]:

(8) $$\frac{\left(\gamma_{tg}-\gamma_{tz}\right)}{\gamma_{tz}}=\cos\theta ,$$

where *θ* is the equilibrium contact angle and *γ* is the interfacial free energy; *t*—solid phase, *g*—gaseous phase, *z*—liquid phase.

**Figure 7:**
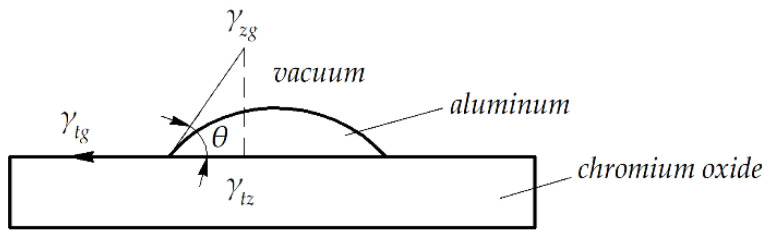
Geometrical representation of the equilibrium of liquid aluminum on the solid surface of chromium oxide.

**Figure 8:**
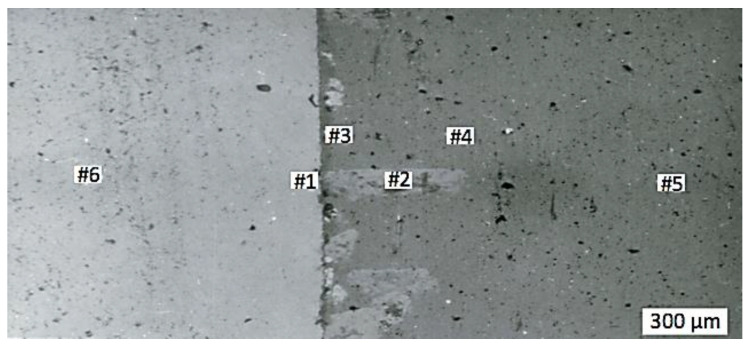
Interface between the aluminum droplet (brighter area on the left) and chromium oxide substrate (darker area on the right) obtained at *T* = 1200 °C and *P* = 10^–2^ MPa. Numbers #1–#6 indicate places where chemical analysis was made.

The equilibrium of liquid aluminum on the solid surface of chromium oxide is presented geometrically in Figure 7, and Figure 8 presents the measurement points in the interface area between the aluminum liquid phase and the chromium oxide substrate, obtained at the temperature of 1200 °C and surrounding pressure *P* of 10^–2^ MPa. Values of the interfacial energy were calculated using the methodology proposed in [22] and are presented in Table 4. Results of the chemical composition measurement are shown in Table 5. Table 6 presents the results of quantitative chemical analysis of points #2 and #5 shown in Figure 8.

It should be noted from Table 4 that the work of adhesion in the system Cr_2_O_3_/Al is higher than that of system Cr_2_O_3_/Cr. The contact angle in the Cr_2_O_3_–Al system is smaller than that of Al_2_O_3_–Cr or Cr_2_O_3_–Cr, both in calculations and in experiments. Chemical analysis (Table 5) suggests that in the phase interface, there is mutual diffusion of chromium and aluminum with prevailing activity of Cr. Thus, due to the good wettability of chromium oxide, the molten aluminum interacts with it during hot pressing, partially restoring chromium.

From Table 6 it can be seen that in the middle of the aluminum droplet (Figure 8), there is a small amount of oxygen, while the amount of diffusing chromium at point #2 was 2.940 wt.% against 0.186 wt.% aluminum at point #5 of the oxide substrate. This can be attributed to the diffusion process between chromium and aluminum ions. 

The light observed phase consists of chromium, while the dark one consists of Cr, Al and O. At increased sintering temperatures, the content of aluminum in the dark phase increases, while that in the light phase decreases. Simultaneously, in the light phase, there is an increase in chromium content and decrease in oxygen content.

Microhardness measurement of the Cr_2_O_3_ + (Cr,Al)_2_O_3_ + Cr cermets demonstrated the dependence of microhardness on the restored chromium amount. This was expected since other studies indicated an increase in Zn-Cr composite coating microhardness with increasing chromium content [37]. This might be the main explanation for the different microhardness values of dark and light phases, which were measured as *HV_d_* = 26 ± 0.15 GPa and *HV_l_* = 12 ± 0.5 GPa, respectively, under the load of 520 g. Moreover, the increased microhardness of chromium itself may be attributed to the additions of aluminum and iron since it is highly dependent on the technology and related additions and thus varies between *HV* = 2 and *HV* = 10 GPa [38]. Noteworthily, nanoindentation revealed the hardness of Cr_2_O_3_ thin films on SiO_2_ glass substrate ranging from 12 to 22 GPa [39].

**Figure 9:**
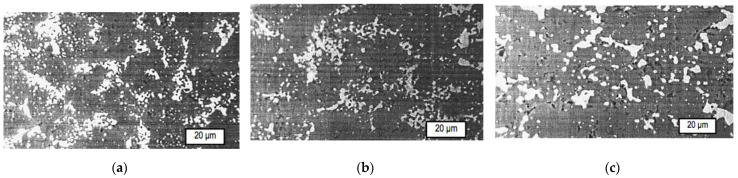
Microstructure of Cr_2_O_3_ + (Cr,Al)_2_O_3_ + 22 wt.% Cr cermet samples sintered from Cr_2_O_3_ + 13 wt.% Al powder at *T* = 1500 °C under *P* = 30 MPA for different lengths of time *t*: (**a**) 5 min; (**b**) 10 min; (**c**) 15 min.

Figure 9 shows microstructure of Cr_2_O_3_ + (Cr,Al)_2_O_3_ + 22 wt.% Cr cermet samples sintered at *T* = 1500 °C under *P* = 30 MPA for different lengths of time *t*. It can be seen that metallic chromium grains are distributed throughout the oxide matrix in the form of a number of clusters of small particles. This may be explained by the unsteady distribution of the liquid aluminum in the sample volume in the initial stage of the hot-pressing process.

**Figure 10:**
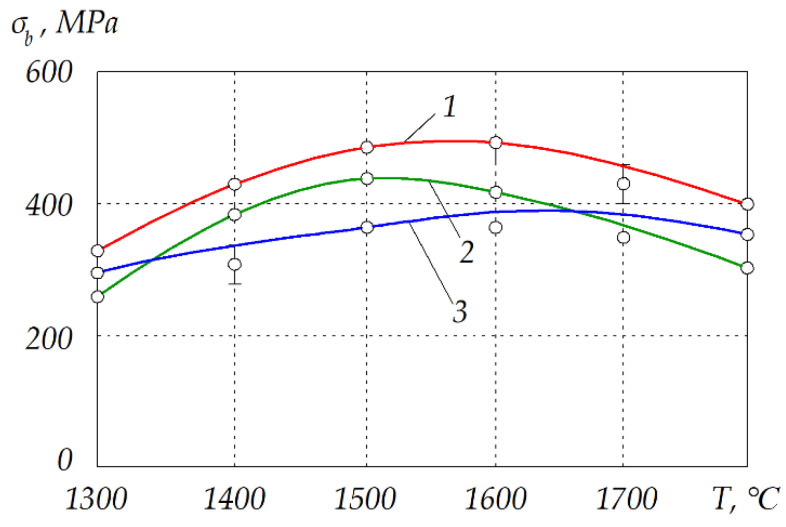
Dependence of flexural strength on sintering temperature of different cermets hot pressed under *P* = 30 MPa for *t* = 10 min: *1*—Cr_2_O_3_ + (Cr,Al)_2_O_3_ + 22 wt.% Cr; *2*—Cr_2_O_3_ + (Cr,Al)_2_O_3_ + 30 wt.% Cr; *3*—Cr_2_O_3_ + (Cr,Al)_2_O_3_ + 15 wt.% Cr.

**Figure 11:**
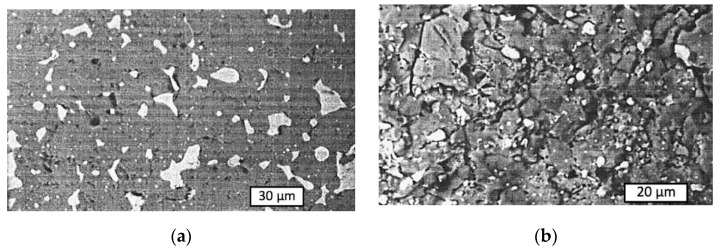
Cermet samples sintered from Cr_2_O_3_ + 13 wt.% Al powder at *T* = 1600 °C under *P* = 30 MPA for *t* = 10 min: (**a**) microstructure; (**b**) fractogram.

The composition of the solid solution Cr*_x_*,Al_2–*x*_O_3_ formed in the phase interfaces depends not only on the initial proportion, but also on the temperature and time of sintering. The dependence of the flexural strength *σ_b_* on sintering temperature is plotted in Figure 10. It should be noted that eutectic proportion with 22 wt.% chromium (red curve #1) exhibited higher flexural strength than other proportions and than typical values for alumina ceramics, which range from 266.7 to 357.5 MPa [40]. The highest strength was obtained for the composition Cr_2_O_3_ + (Cr,Al)_2_O_3_ + 22 wt.% Cr at *T* = 1600 °C after *t* = 10 min, perhaps as a result of the strongest bond between the phases that correspond with an equimolar composition of (Cr,Al)_2_O_3_. The structure and fractogram of this cermet are presented in Figure 11.

It can be assumed that the submicron dimensions of the grains seen in Figure 9 and Figure 11 contributed to the improvement of the composite characteristics. As indicated by other studies, nanometric particles of materials, including Cr_2_O_3_, usually provide physical and mechanical properties superior to those of conventional particles [41].

**Figure 12:**
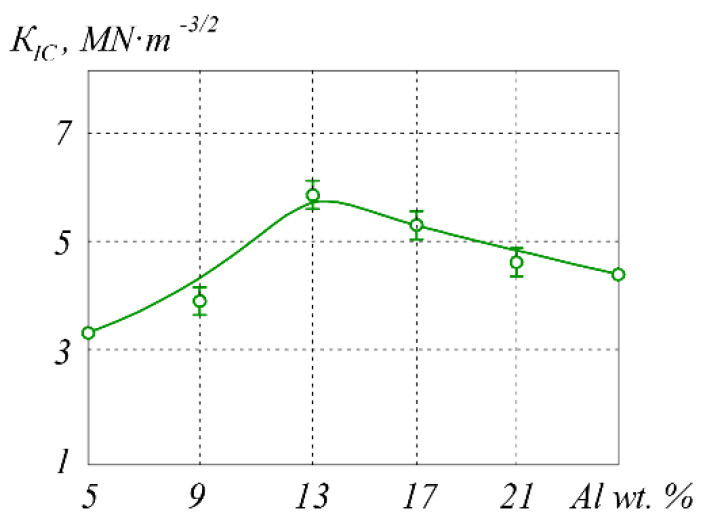
Fracture toughness of cermet samples sintered from initial powders of Cr_2_O_3_ + Al with different proportions of aluminum at *T* = 1600 °C under *P* = 30 MPA for *t* = 10 min.

**Figure 13:**
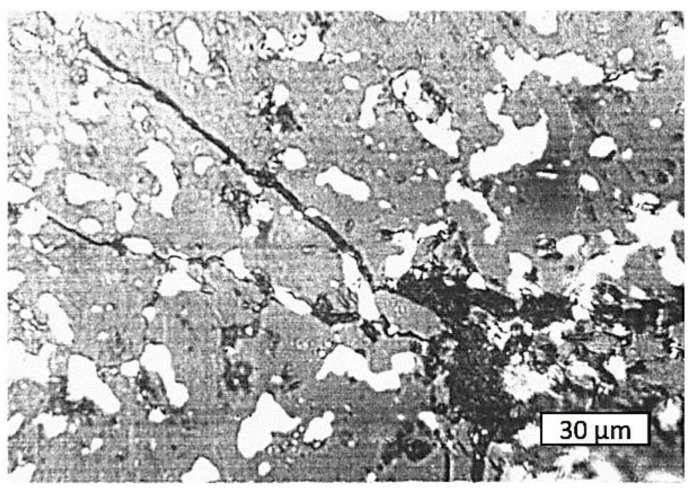
Crack propagation in the Cr_2_O_3_ + (Cr,Al)_2_O_3_ + 22 wt.% Cr cermet sample sintered at *T* = 1600 °C under *P* = 30 MPA for *t* = 10 min.

Fracture toughness results for different aluminum contents are presented graphically in Figure 12. The comparative analysis revealed that the highest value of *K_IC_* can be obtained for the Cr_2_O_3_ + 13 wt.% Al proportion of the initial powders, which resulted in the desired Cr_2_O_3_ + (Cr,Al)_2_O_3_ + 22 wt.% Cr composition of the cermet. Crack propagation in its structure is illustrated in Figure 13.

From the microstructure seen in Figure 13, it can be concluded that the fracture toughness is increased due to the presence and dimensions of the grains of chromium in the matrix. The deflection of cracks bypassing the light phase grains obviously contributed to the toughening of the composite, while strong interface bonds determined its strength. This is consistent with other published studies indicating that weak interfaces are detrimental to composite strength and that the matrix carries a part of the load when the interfaces are strong [42]. Crack deflection is considered in toughening models [43] since it simply changes the value of *K_IC_* compared to the case of straight crack propagation. The crack deflection mechanism is related to the changes in applied stress and its reduction in the crack front, which require high energies for cracks to achieve critical size [44].

## 5. Conclusions

The investigations in this study were focused on the hot-pressing process of chromia-based cermets. The ability of the sintering method to produce a eutectic composition of chromia and alumina was demonstrated through the mechanism of an aluminothermic reaction. The eutectic composition with 22 wt.% restored chromium was successfully sintered from a Cr_2_O_3_ + 13 wt.% Al initial powder mixture. During the reaction, chromium was restored, providing cermets of different chemical compositions. It was found that due to the presence of aluminum powder, full densification could be reached at 1600 °C after 2–3 min. On the other hand, the sintering in temperature interval between 1300 °C and 1400 °C under the pressure of 30 MPa would result in full completion of the restoration reaction and thus no pure aluminum content. It was found that during the hot pressing, aluminum melted, introducing a liquid phase to the sintered powders, which served as a sintering aid for the ceramic matrix composite.

Another important mechanism supporting the fabrication of the chromia-based composite was the appearance of the solid solution. Unlike the cermets obtained from mixtures of chromium and chromium oxide, the tested samples with added aluminum contained a solid solution of (Cr,Al)_2_O_3_ in the interfaces between the phases after sintering. Solid solution Al_2–*x*_Cr*_x_*O_3_ with varying stoichiometric composition had a strong effect on the boundaries between phases and thus on the overall mechanical strength of the composite. The tested cermets exhibited differentiation of properties, mainly determined by the metal and ceramic characteristics, the volume proportion between them and the adhesion energies, but also by the initial powders, their morphology (particle dimensions and shape) and sintering conditions. Sintering conditions have a direct impact on the stoichiometric composition of the solid solution in the phase interface which determines the mechanical properties of the cermet composite.

The highest strength and toughness were reached for the eutectic composition Cr_2_O_3_ + (Cr,Al)_2_O_3_ + 22 wt.% Cr, where 22 wt.% was restored chromium. Sintered at *T* = 1600 °C under *P* = 30 MPa for *t* = 10 min, it exhibited flexural strength *σ_b_* = 490 MPa and fracture toughness *K_IC_* = 5.8 MN/m^3/2^. These characteristics are advantageous for ceramic cutting tools, so this seems to be a promising research direction. Further investigations will provide comparative characteristics of the obtained cermet and typical alumina-based materials of cutting tools, as well as an analysis of the ways for further improvement.

## Figures and Tables

**Table 1:** Calculated equilibrium concentration *C_e_* in mol/L of components in the cermet obtained by hot pressing of powders Cr_2_O_3_ + 13 wt.% Al under different pressure *P* and temperature *T* conditions.

*T*, °C	Components	*P =* 30 MPa	*P =* 0.1 MPa	*P =* 0.01 MPa
700	Cr_2_O_3_	1.217	1.055	1.055
	Al_2_O_3_	3.917	0.407	4.077
	Cr	7.835	8.153	8.158
	Al	0.001	–	–
800	Cr_2_O_3_	2.385	1.055	0.087
	Al_2_O_3_	0.052	4.077	4.843
	Cr	8.104	8.153	9.687
	Al	0.049	–	–
900	Cr_2_O_3_	1.055	1.055	1.055
	Al_2_O_3_	4.077	4.077	4.077
	Cr	8.153	8.153	8.153
	Al	–	–	–
1000	Cr_2_O_3_	1.055	1.055	1.055
	Al_2_O_3_	4.077	4.077	4.077
	Cr	8.153	8.153	8.158
	Al	–	–	–
1100	Cr_2_O_3_	0.721	0.621	0.621
	Al_2_O_3_	1.368	0.319	0.319
	Cr	4.346	6.214	6.214
	Al	1.030	–	–
1200	Cr_2_O_3_	0.965	0.621	0.621
	Al_2_O_3_	1.063	0.319	0.319
	Cr	5.605	6.214	6.214
	Al	0.065	–	–
1300	Cr_2_O_3_	1.217	0.422	0.422
	Al_2_O_3_	0.698	0.915	0.915
	Cr	6.713	7.215	7.215
	Al	–	–	–
1400	Cr_2_O_3_	1.217	0.422	0.422
	Al_2_O_3_	0.698	0.915	0.915
	Cr	6.713	7.215	7.215
	Al	–	–	–

**Table 2:** Lattice parameters *a* and *c* for the sintered powders with different aluminum contents.

No.	Initial Powder Content	*a*_1_, Å	*a*_2_, Å	*a*_3_, Å	*c*_1_, Å	*c*_2_, Å	*c*_3_, Å
1	Cr_2_O_3_ + 9 wt.% Al	4.947	4.936	4.922	13.302	13.366	13.433
2	Cr_2_O_3_ + 13 wt.% Al	4.941	4.936	4.922	13.366	13.410	13.433
3	Cr_2_O_3_ + 17 wt.% Al	4.936	4.926	4.922	13.410	13.428	13.433

**Table 3:** Percentage by weight of Al and Cr in the samples of Cr_2_O_3_ + (Cr,Al)_2_O_3_ + 22 wt.% Cr sintered at *T* = 1400 °C and *P* = 30 MPa for different lengths of sintering time *t*.

Compound	*t =* 5 min	*t =* 10 min	*t =* 15 min
Aluminum	40.5	27	13.5
Chromium	26	52	78

**Table 4:** Contact angles and energy characteristics of the MeO–Me systems.

System	*T*, K	Adhesion Work *W_a_* and Its Components, MJ/m^2^				*θ, °*	
		*W_ch_*	*W_b_*	Calculated	Experimental	Calculated	Experimental
Al_2_O_3_—Al	1500	510	243	753	1260 ± 50	98	60
Al_2_O_3_—Cr	2173	0	285	285	2260 ± 50	145	65
Cr_2_O_3_—Al	1573	730	235	955	1400 ± 50	118	60
Cr_2_O_3_—Cr	1573	605	260	865	1360 ± 50	120	65

**Table 5:** Content of chromium and aluminum at the respective points of MeO–Me interfaces.

Content, wt.%					
#1	#2	#3	#4	#5	#6
Cr/Al	Cr/Al	Cr/Al	Cr/Al	Cr/Al	Cr/Al
99.161/0.423	45.946/53.706	13.707/85.856	7.197/92.475	6.027/93.761	98.917/0.339

**Table 6:** Quantitative analysis of composition at the respective points of MeO–Me interfaces.

Content, wt.%					
#6			#5		
Cr	Al	O	Cr	Al	O
2.940	95.776	1.329	96.195	0.186	3.414

## Data Availability

Data available on request due to privacy restrictions.
